# Development of a Simple Electroless Method for Depositing Metallic Pt-Pd Nanoparticles over Wire Gauge Support for Removal of Hydrogen in a Nuclear Reactor

**DOI:** 10.3390/ma16196541

**Published:** 2023-10-03

**Authors:** Kiran K. Sanap, Sawanta S. Mali, Deepak Tyagi, Ajit N. Shirsat, Suhas B. Phapale, Suresh B. Waghmode, Salil Varma

**Affiliations:** 1 Shri Guru Gobind Singhji Institute of Engineering and Technology, Nanded 431606, India; 2 Chemistry Division, Bhabha Atomic Research Centre, Trombay, Mumbai 400085, Indiasuhaschemistry@gmail.com (S.B.P.); svarma@barc.gov.in (S.V.); 3 Polymer Energy Materials Laboratory, School of Chemical Engineering, Chonnam National University, Gwangju 61186, Republic of Korea; sawanta@jnu.ac.kr; 4 Homi Bhabha National Institute, Mumbai 400094, India; 5 Department of Chemistry, Savitribai Phule Pune University, Pune 411007, India; suresh.waghmode@gmail.com

**Keywords:** electroless deposition, platinum, palladium, formaldehyde, hydrogen mitigation

## Abstract

Electroless noble metal deposition on the conducting substrate is widely used to obtain the desired film or coating on the substrate of interest. Wire-gauge-based Pt/Pd/Pt-Pd (individually, sequentially, and simultaneously deposited) catalysts have been developed using formaldehyde and sodium formate as reducing agents. Various surface pretreatment methods like SnCl_2_ + PdCl_2_ seeding, oxalic acid etching, and HCl activation (etching) have been employed to obtain the desired noble metal coating. Minimum time duration was observed for simultaneously deposited catalysts using formaldehyde as a reducing agent. Prepared catalysts were characterized for noble metal deposition, coating kinetics, surface morphology, and binding energy. The catalyst was found to be active for H_2_ and O_2_ recombination reactions for hydrogen mitigation applications in nuclear reactors.

## 1. Introduction

In modern research, electroless metal plating techniques are widely used in several fields of science and technology, including metallization, galvanoplastic, microcircuits, and optoelectronics. Additionally, they are effectively employed to create catalysts for fuel cells as well as in several catalytic processes, including catalytic steam methane reforming (SMR), methanol oxidation in direct methanol fuel cells, etc. [1,2,3,4,5,6,7,8,9,10]. These include thermal deposition [11,12], sonication [13,14], UV irradiation [15,16], microwave [17,18], sputter coating [19,20], chemical vapor deposition [21,22], electrochemical plating [23,24] and electroless plating [25,26], etc. Although thermal deposition, sputter coating, and chemical vapor deposition provide excellent control over film composition, thickness, and structure, they suffer from high cost (expensive high vacuum equipment is required) and low yield. Electrochemical plating is only suitable for conducting substrates. Electroless plating provides uniform dense coatings on both conducting and non-conducting surfaces [27]. It is also a relatively inexpensive process.

Noble metal electroless deposition is an electrochemical process that is heterogeneous, autocatalytic, and redox in nature. The interaction between the reducing agents from the electroplating bath and the noble metal precursors on the surface of the metal substrate takes place at the interface between solid and liquid. The procedure can be carried out at different temperatures and pH levels, with or without the addition of stabilizing or complexing agents, depending on the type of coating required and its uses. The coating process was carried out at room temperature with or without the use of a complexing agent to meet the demand for crystalline or amorphous noble metal coating. 

Deposits/thin films obtained from electroless deposition have many applications in different fields. The primary application is the protection of the underlying layers against corrosion [28,29]. Other applications include wear resistance for surfaces, ornamental coatings to improve the aesthetic appeal of objects and practical applications such as providing catalytic surfaces for electrodes used in chemical processes and low-resistance electrical connections. Metals with high melting points, such as platinum and rhodium, are primarily deposited using the electroless deposition method. One of the vast applications of these electroless deposited catalysts is in the field of hydrogen mitigation. In nuclear reactors, the catalytic mitigation of hydrogen is one of the most promising methods to keep hydrogen concentration below the combustion limit. For these purposes, wire-gauge-supported Pt/Pd catalysts prepared using the electroless deposition method found extensive use for the recombination of H_2_ and O_2_ in a nuclear reactor [30,31,32,33,34]. In this article, we report the development of an electroless deposition method for the preparation of Pt-based, Pd-based, and Pt-Pd (sequential and simultaneous) catalysts by employing sodium formate and formaldehyde as the reducing agents. It has been noted previously that the presence of complexing agents causes a bright metallic covering that is ineffective as a catalyst [35]. Depending on the type of coating required and its uses, the coating procedure can be carried out at different temperatures and pH levels, with or without the addition of stabilizing or complexing agents [36]. In this article, we report room-temperature deposition of metallic Pt-Pd nanoparticles over a wire gauge support without the use of a complexing agent, without alteration in pH, and without using any inert gas bubbling (Argon) or agitation to meet our demand for an amorphous noble metal coating with improved surface heterogeneity. Prepared catalysts were characterized for % noble metal deposition and coating kinetics using UV-Vis spectrophotometry. Further, an investigation for the study of metal–metal or metal–support interaction was carried out by XPS. Finally, the catalysts were evaluated for H_2_ and O_2_ recombination reactions.

## 2. Experimental

### 2.1. Electroless Deposition

In the present study, Hydrochloric acid (Thomas Baker, Mumbai, India, 36%) and Oxalic acid (Chemico Fine Chemicals, Delhi, India, 99.8%) were used as etching agents for the substrate. Tin Chloride (Loba Chemie, Mumbai, India, 98%) was used as a seeding agent. Sodium formate (S D Fine Chemical, Mumbai, India) and formaldehyde (Thomas Baker, Mumbai, India, 37–41%) were employed as reducing agents. Chloroplatinic acid (Hindustan Platinum Private Limited, Mumbai, India, 39.8% Pt) and palladium chloride (Hindustan Platinum Private Limited, Mumbai, India, 59.96% Pd) were employed as noble metal precursors and an SS-316 wire gauge with wire dimensions of 0.1 mm and mesh size of 80 as a substrate for deposition. The concentrations of precursor solutions used in the present study were 3.6 mg/mL or 12 mg/mL of Pd and 9.5 mg/mL of Pt. The coating procedure relies on several processes which occur at the metal support surface, including the oxidation of formaldehyde at the anode surface and the reduction of noble metal at the cathode surface [37].

**Figure 1:**
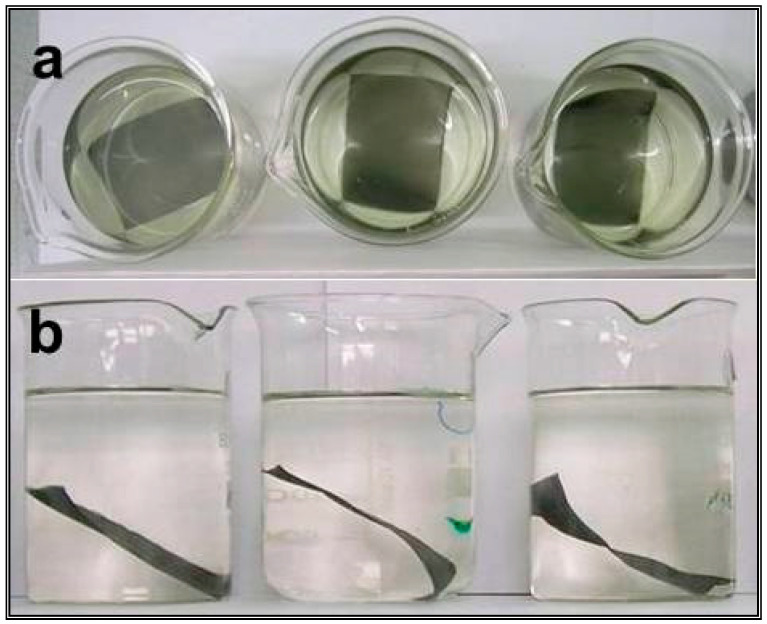
Photographs for electroless reduction (**a**) before deposition and (**b**) after deposition of noble metal on 6 cm × 8 cm wire gauge support.

A study of electroless deposition and catalytic activity evaluation was carried out over 4 cm × 6 cm and 6 cm × 8 cm dimensions of an SS wire gauge. First, 4 cm × 6 cm (or 6 cm × 8 cm) washed wire gauge pieces were subjected to HCl etching and then dipped in an electroless deposition bath. Different activation processes like electrolytic oxalic acid etching, SnCl_2_ + PdCl_2_ seeding, etc., were also employed. In general, the electroless deposition bath consisted of noble metal precursors like palladium chloride or chloroplatinic acid along with reducing agents like formaldehyde or sodium formate. The time required for the coating to complete was found to depend on the concentration of the noble metal precursor and/or reducing agent. Depending on the composition and other experimental conditions, shiny to amorphous coatings were observed. Various compositions with single or mixed noble metals were prepared. A typical electroless deposition method is shown in Figure 1a,b.

### 2.2. Catalytic Activity

Using a thermal conductivity detector-based H_2_ monitor, the prepared catalysts were assessed for catalytic H_2_-O_2_ recombination reaction under static air reaction conditions in a 40 L closed reactor at room temperature (RT). The time required for completion of half of the reaction (t_1/2_) and maximum temperature rise on the catalyst surface (T_max_) values for the H_2_-O_2_ reaction at a 4.5% H_2_ concentration in air were assessed. Details of catalytic activity setup were given in an earlier article reported by Sanap et al. [30].

## 3. Results and Discussion

### 3.1. Catalyst Preparation 

Effective noble metal deposition on a catalyst substrate is widely influenced by the reducing agent and pre-treatment method used for activation of the substrate. The following section elaborates on the effect of the catalyst preparation route adapted and its effect on the noble metal deposition and catalytic activity.

#### 3.1.1. Palladium on SS Wire-Gauge-Based Catalysts

The wire gauge obtained from the industry had a smooth texture and additionally, it was composed of dirt, oil, grease, etc. To obtain a good quality of coating, it was necessary to remove all contaminants from the surface. Different methods have been reported in the literature to make wire gauge surfaces clean and rough, e.g., HCl etching^,^ [38], SnCl_2_ + PdCl_2_ seeding [39], electrolytic oxalic acid activation, etc. [40]. 

Work on the preparation of a Pd-supported catalyst using HCl etching was initiated from the reported process with Na-formate as a reducing agent [41]. For this purpose, initially cleaned and dried wire gauge pieces were pre-treated with 0.1 N HCl for 15 h. Afterward, they were taken out, washed with plenty of distilled water, and dried in an oven at 105 °C for 1 h. Then, the wire gauge coupons (4 cm × 6 cm) were kept in an electroless plating bath (125 mL beaker) containing a 100 mL diluted solution of precursor and reducing agent. The procedure adopted and the catalytic activity of the prepared catalysts are given below in Table 1. Thus, e.g., bath A1 consisted of 2.5 mL of PdCl_2_ and 1 mL of Na-formate which were then further diluted to 100 mL. After 15 h of deposition, no weight gain or catalytic activity were observed for the catalysts prepared by the direct deposition route using HCl etching treatment. Therefore, another additional surface activation step using SnCl_2_ + PdCl_2_ activation was introduced after HCl etching for the more effective deposition of noble metal. This process of activation involved dipping a wire gauge for 5 min each in SnCl_2_ (1 gm/L), distilled water, PdCl_2_ (0.1 gm/L) solution, and 0.01 M HCl. This procedure was repeated 4–10 times with intermittent rinsing with distilled water. This was followed by electroless deposition using Na-formate as a reducing agent as per the procedure given for HCl-etched catalysts. The bath was found to turn turbid after 2–3 h and the catalysts prepared were also found to be inactive for the H_2_-O_2_ recombination reaction. Catalysts prepared by this route did not show any weight gain and were also found to be catalytically inactive. Finally, another option for electrolytic oxalic acid etching (wire gauge: cathode + ve, potential: 5 V, current: 0.1 A, time: 40 s) was introduced before electroless reduction. In this case, limited noble metal coating was obtained but the prepared catalysts did not exhibit the required catalytic activity.

In the case of sodium formate using SnCl_2_ + PdCl_2_ or oxalic acid activation, additional efforts were made for pH adjustment, but the bath was still found to turn turbid with time. Also, no activity was observed for the prepared catalysts. 

Based on the above observations (i.e., failure of resultant noble metal deposition on the substrate), a different reducing agent, i.e., formaldehyde, was employed for further study. This was based on an earlier reported similar procedure for platinum coating [42]. Different activation steps like HCl etching, SnCl_2_ + PdCl_2_ seeding, and electrolytic etching using oxalic acid were employed as pre-treatment steps. Details are provided in Table 2.

From the data given in Table 2, it is evident that catalysts prepared using formaldehyde as a reducing agent showed the deposition of noble metal on the substrate, but catalytic activity was observed only for the catalysts that were produced after electrolytic etching in oxalic solution. Therefore, the process based on electrolytic etching followed by noble metal reduction using formaldehyde was short-listed for further studies on larger (6 cm × 8 cm) catalysts. During this process, wire gauge coupons of 6 cm × 8 cm were rotated (1 RPM) with the help of a motor in a coating bath (500 mL beaker) containing a 400 mL diluted solution of precursor and reducing agent. The details of the samples prepared are provided in Table 3. During the coating of palladium, the color of the bath was found to change from yellowish or yellowish orange to nearly colorless as coating took place on the substrate. The coating observed was uniform, black, smooth, fine, and had good adherence. All the catalysts prepared had a weight gain of ~2 wt % and exhibited good catalytic activity for the H_2_-O_2_ reaction, with t_1/2_ ranging from 3–3.5 min and T_max_ of 200–300 °C.

#### 3.1.2. Platinum on SS Wire-Gauge-Based Catalysts

Details of the Pt-based catalysts prepared and their catalytic activity data are listed in Table 4. During this process, wire gauge coupons of 6 cm × 8 cm were rotated with the help of a motor in a coating bath (500 mL beaker) containing a 400 mL diluted solution of precursor and reducing agent. During the coating of platinum, the bath color changed from yellowish or pale yellowish to colorless. The coating observed was uniform, smooth, and blackish-white without any dark patches on the wire gauge and had good adherence. Weight gain for the noble metal coating was found to vary from 1.5 to 2.0 wt %. Catalytic activities in terms of t_1/2_ and T_max_ were found to be about 5.0 min and 245–275 °C, respectively. The inorganic acid-etched catalysts D1 and D3 (HCl-etched) were found to be superior to the organic acid-etched catalysts D2 and D4 (oxalic acid-etched). Coating took place in a short time and t_1/2_ was also comparable. This may be due to the strength of the acid and pH of both solutions. The coating was also smooth, fine, uniform, and had a black amorphous type with good adherence. Thus, for platinum-based catalysts, it was observed that activation of the SS wire gauge with 0.1 N HCl is sufficient for the electroless deposition procedure.

#### 3.1.3. Mixed Noble Metal Deposition on SS Wire-Gauge-Based Catalysts

Further efforts were directed towards the development of a procedure for the deposition of mixed noble metals (Pt and Pd) on an SS wire gauge. For this purpose, three approaches were adopted: firstly, a sequential deposition of palladium over platinum. The second approach was platinum over palladium, but in this case, we observed that the process was too tedious, i.e., for the initial deposition of Pd, electrolytic oxalic acid etching was necessary, as observed in Table 2. Additionally, it is very time-consuming if we consider mass scale preparation of catalysts. Finally, simultaneous deposition of both platinum and palladium was carried out in a single electroless bath. Table 5 shows the details of sequentially (E) and simultaneously (F) prepared catalysts using formaldehyde as a reducing agent over 6 cm × 8 cm SS wire gauge support. 

For the sequential noble metal deposition, it was observed that the coating process was very slow, taking almost ~20 h (10 + 10) for ~2% weight gain (E1, E2, and E3 samples). Catalysts produced were found to be active for H_2_ + O_2_ reaction with t_1/2_ = ~2.5–2.8 min and T_max_ = ~295 °C.

To minimize the time of coating, we tried simultaneous deposition using HCl etching and oxalic acid etching treatment for SS support (F1, F2, and F3). In this case, coating time was observed to be only 9 h, and catalytic activity was observed only for those samples (F2 and F3) that were prepared via the HCl activation route.

The results obtained from the above study show that better noble metal deposition was observed when using formaldehyde as a reducing agent rather than sodium formate. For the individual platinum and simultaneous noble metal (Pt-Pd) deposition on the SS wire gauge, activation (etching) by (0.1 N) HCl was more appropriate than using oxalic acid and SnCl_2_ activation. Therefore, we recommend the use of formaldehyde as a reducing agent and hydrochloric acid for surface activation for further detailed study.

### 3.2. Coating Kinetics

**Figure 2:**
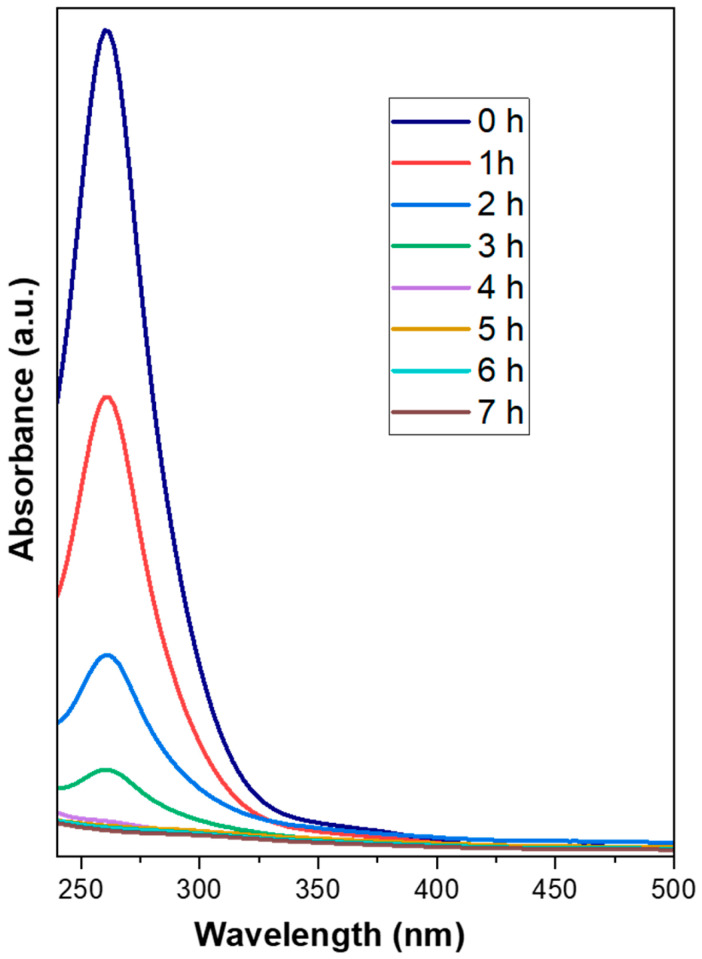
Plot of absorbance of F3 catalyst.

**Figure 3:**
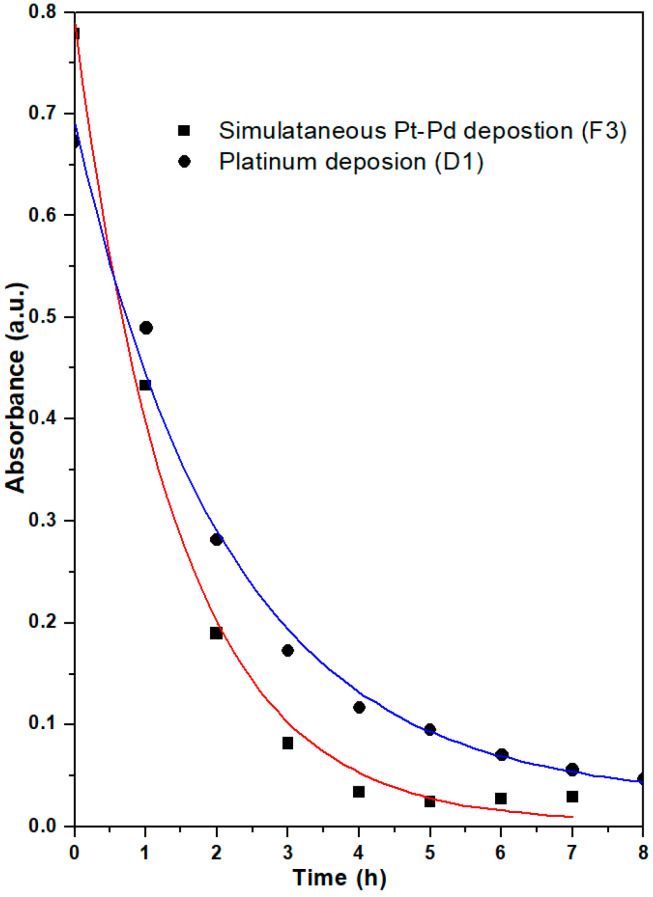
Plot of absorbance against deposition time for F3 and D1 catalysts at λ_max_ = 260.

The deposition of precursors was carried out with the electroless deposition method using formaldehyde as a reducing agent. Both precursor solutions (chloroplatinic acid and palladium chloride) have particular absorbance spectra; therefore, it is possible to interpret their rate of deposition using UV-VIS spectrophotometry. Figure 2 shows the absorbance spectra of the electroless deposition bath of simultaneously prepared Pt-Pd (F3) catalyst with the time of deposition. The absorbance spectrum corresponds to the hexachloroplatinic acid and palladium chloride precursors. It can be observed from the spectrum that the initial concentration of the deposition bath concerning chloroplatinic acid and palladium chloride fell with increasing deposition time. Figure 3 shows the decay plot of absorbance with time for the simultaneously prepared Pt-Pd catalyst and Pt-based catalysts in an electroless deposition bath. From Figure 3, it is apparent that the rate of deposition is quicker for a bi-metallic (Pt-Pd) precursor bath than for a single-metal (Pt) precursor bath. Here it is also evident that for both the catalysts, the initial rate of coating is slow for up to 1 h, and after 1 h it becomes quicker but then becomes slower again after 3 h. This may be due to the following reasons: initially, the even distribution of smaller nuclei is taking place on the substrate. These smaller nuclei act as catalytic agents for further processes and hence the method becomes autocatalytic. After 3 h, the quantity of reactants starts diminishing in the electroless deposition bath and the rate of coating again further falls.

**Figure 4:**
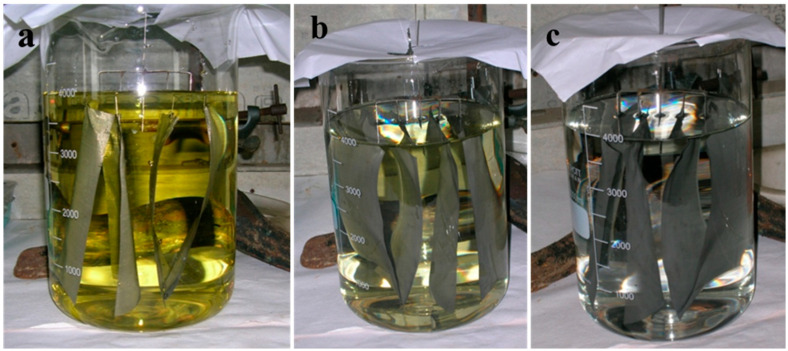
Photographs for electroless reduction for F3 catalyst (**a**) at the start of deposition, (**b**) at 2 h of deposition, and (**c**) at 6 h of deposition of noble metal on 12 cm × 16 cm wire gauge support.

Figure 4 shows that at the start of deposition, the noble metal deposition bath has a dark yellow color which becomes lighter as further coating takes place on the wire gauge.

### 3.3. Surface Morphology

**Figure 5:**
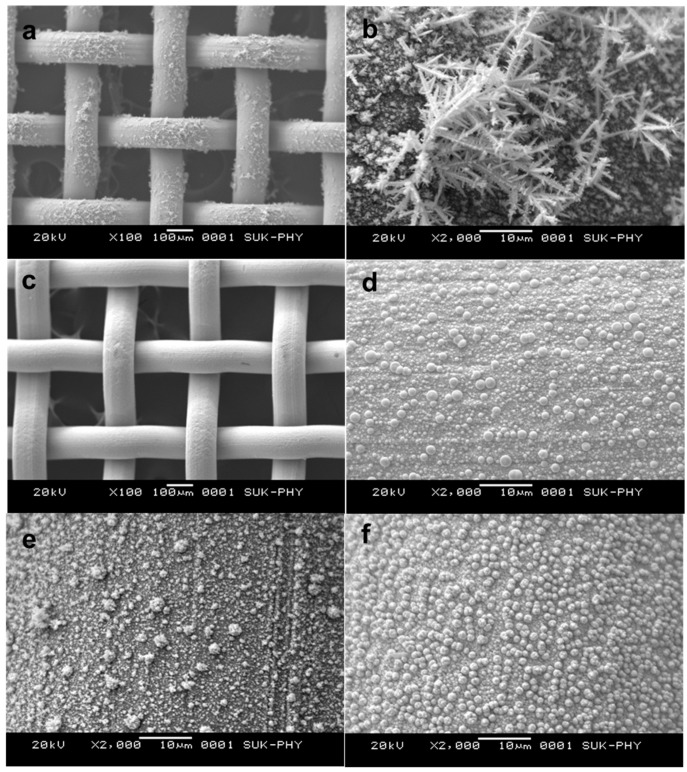
Scanning electron micrograph of (**a**,**b**) Pd-deposited sample (A9) using Na formate as reducing agent, (**c**,**d**) Pt-deposited sample (D1), (**e**) Pt-Pd sequentially deposited sample (E2), and (**f**) Pt-Pd simultaneously deposited sample (F3) using formaldehyde as a reducing agent.

Scanning electron microscopy was employed to understand the noble metal deposition using different reducing agents and surface activation methods. Figure 5a,b show the palladium-deposited sample using sodium formate as the reducing agent. Figure 5a shows the nonuniform coating of the noble metal on the surface of the wire gauge. Figure 5b depicts the precipitation of the noble metal precursor on the surface of the wire gauge. This precipitated noble metal resulted in the loss of catalytic activity for this catalyst. Figure 5c,d pertains to the uniform coating of Pt noble metal (sample D1) on the surface of a wire gauge using formaldehyde as a reducing agent. Here, deposited noble metals are observed with perfect round and spherical geometry. Figure 5e shows the accumulation of palladium particles on the initially deposited platinum which has initiated, resulting in the formation of spike geometry on the earlier deposited Pt metal (Sample E2). Figure 5f pertains to the Pt-Pd simultaneously deposited sample (F3) using formaldehyde as a reducing agent. Here, particles are observed with well-defined cuboidal extension to the spherical particles. 

### 3.4. X-ray Photoelectron Spectroscopy

**Figure 6:**
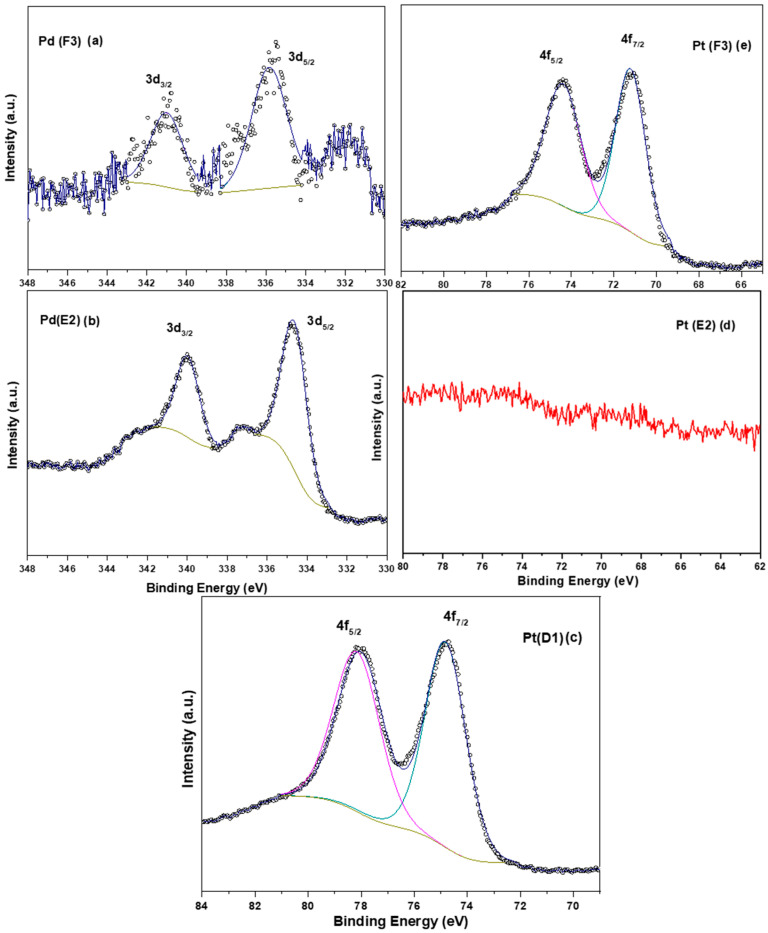
(**a**) XPS spectra of Pd 3d region for F3 sample, (**b**) XPS spectra of Pd 3d region for E2 sample, (**c**) XPS spectra of Pt 4f region for D1 sample, (**d**) XPS spectra of Pt 4f region E2 samples, and (**e**) XPS spectra of Pt 4f region for F3 samples.

X-ray photoelectron spectroscopy (XPS) analysis was performed for Pt-based (D1), Pt-Pd sequentially prepared (E2), and simultaneously prepared Pt-Pd (F3) samples. All three samples, D1, E2, and F3, were analyzed for Pt, whereas E2 and F3 were analyzed for Pd alone. The peaks corresponding to Pt for D1 and F3 samples were deconvoluted into two peaks, i.e., 4f_5/2_ and 4f_7/2_ with the respective binding energy of ~74 and 71 eV (Figure 6c,e). The observed binding energy for Pt (4f_7/2_) in D1 and F3 samples is 71.4 and 71.2 eV. These values are in good agreement with the values of Pt^0^ reported in the literature [43,44]. Hence, our study shows that the noble metals were in the metallic state. No characteristic peak (Figure 6d) was observed for Pt in the E2 sample; this could be because of the external coating of Pd on an existing layer of Pt.

For the analysis of Pd concerning the F3 and E2 samples (Figure 6a,b), the spectrum was deconvoluted into two peaks, 3d_5/2_ and 3d_3/2_, with respective binding energy of ~335 eV and ~340 eV. The 3d_5/2_ binding energy contribution of Pd (F3 sample) was 335.8 eV, whereas for the E2 sample it was 334.8 eV. Both values resemble the presence of Pd in a metallic state (Pd^0^) [45], although a slight shift (+1 eV) is observed in binding energy for Pd in the case of the F3 sample [46]. The shift in binding energy can be explained based on the procedure of coating. The coating in F3 is carried out simultaneously so there is a possibility of homogenous solid solution formation and earlier deposition of platinum, whereas the coating of the E2 sample is carried out sequentially and therefore there is only a deposition of Pd on the existing Pt, resulting in no resultant shift in binding energy value. Therefore, in the F3 sample, electrons can transfer more easily from Pt to Pd or vice versa. The inference drawn from the XPS data supports the earlier investigation of the initial coating of platinum later followed by palladium in the case of the F3 sample.

### 3.5. Catalytic Activity

**Figure 7:**
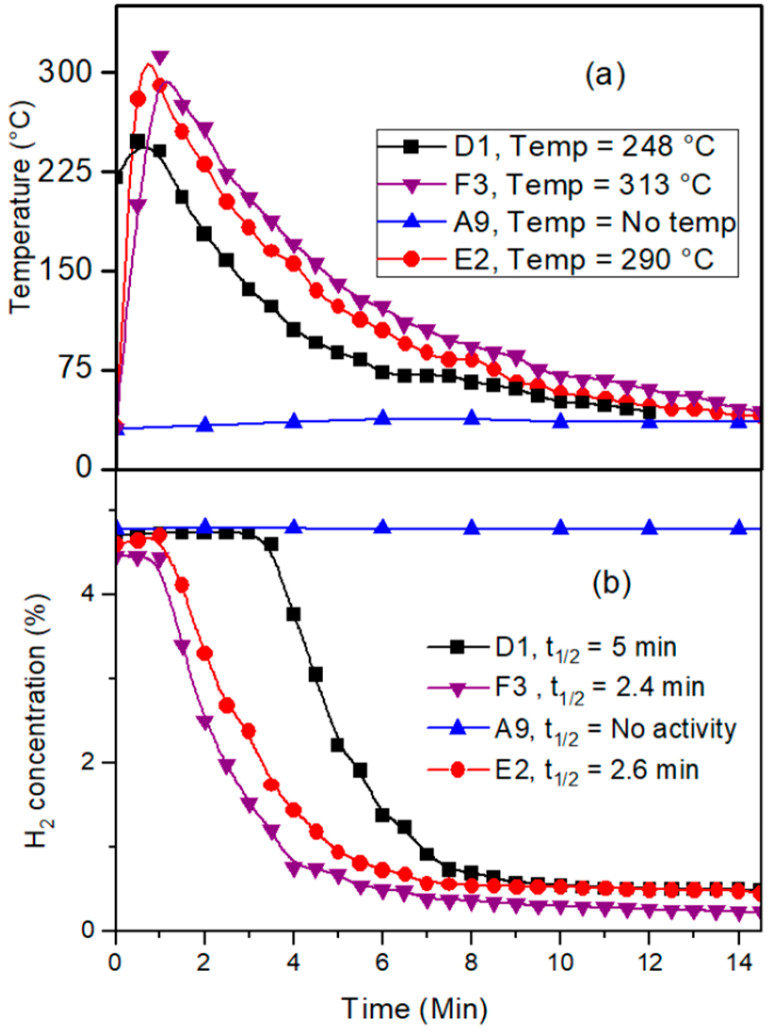
(**a**) Change in catalyst temperature with time, (**b**) Variation in catalytic activity concerning H_2_ concentration for D1, E2, A9, and F3 catalysts.

In a 40 L closed-vessel reactor, the prepared catalysts’ catalytic activity for the reaction of hydrogen and oxygen was assessed. A fixed-volume injector was used to inject a known amount of hydrogen into a reactor, and the hydrogen concentration and surface temperature of the catalyst were used to track the reaction’s progress. Figure 7a shows the variation in temperature on the catalyst surface and Figure 7b shows the variation in catalytic activity concerning the noble metal deposition method adapted. The palladium-based sample (A9) prepared with the pretreatment of oxalic acid and Na-formate as a reducing agent showed no catalytic activity because of the precipitation of the noble metal precursor on the surface of the wire gauge. This result goes hand in hand with the result obtained from the surface morphology study. The platinum-based sample (D1) prepared by HCl pretreatment and formaldehyde as a reducing agent showed t_1/2_ = 5 min and the catalyst temperature was observed to be around 250 °C. Round and spherical particles of Pt were observed to be catalytically active toward H_2_ and O_2_ reaction. Bimetallic Pt-Pd-based (E2 and F3) samples prepared by sequential deposition and simultaneous deposition showed t_1/2_ in the range of 2.5 min and catalyst surface temperature was observed to be around 290 °C to 313 °C. For these catalysts, a high-temperature rise was observed concerning a single Pt-based catalyst because of the synergic effects of both metals. This high temperature obtained is highly advantageous to keep catalyst poisons (e.g., H_2_O, reaction product) away from the catalyst surface. From the surface morphology study, it was revealed that Pt-based catalysts (D1) were perfectly round in shape, whereas catalyst F (simultaneously prepared bimetallic catalyst) showed surface heterogeneity. The reduced catalytic activity for the catalyst was because of heterogeneity in the surface morphology as compared to the smooth surface of a Pt-based catalyst. Higher surface heterogeneity resulted in the availability of more catalytic active sites on the catalyst surface, which resulted in lower catalytic activity.

## 4. Conclusions

By using a simple electroless deposition method, an effective noble metal deposition method has been developed. Formaldehyde is a superior reducing agent compared to Na-formate. To obtain the desired catalytically active noble metal coating, HCl activation (etching) was found to be more efficient than SnCl_2_ + PdCl_2_ and oxalic acid activation. Simultaneous mixed noble metal (platinum–palladium) catalysts were effectively prepared using formaldehyde as a reducing agent. The rate of the coating was found to be slower at earlier and later stages of deposition in the case of both single Pt-based and mixed noble metal (F3) catalysts. The rate of coating was found to be faster for the simultaneously prepared catalyst (F3) as compared to that of individual noble metal (D1) catalysts. All catalysts, D1, E2, and F3, were observed to be of a metallic nature from the XPS study. Catalysts with surface heterogeneity were found to be more active for H_2_ and O_2_ reactions as compared to the catalysts with smooth and spherical surfaces. For further mass-scale synthesis of catalysts to be used in the passive autocatalytic recombiner, simultaneous deposition of Pt and Pd with formaldehyde as a reducing agent and hydrochloric acid as an etching agent has been suggested, considering lower deposition time and better coating characteristics.

## Figures and Tables

**Table 1:** Details of palladium-based catalysts over 4 cm × 6 cm SS wire gauge coupons using sodium formate as a reducing agent.

Catalyst No	Activation	PdCl_2_ (mL) (Pd = 3.6 mg/mL)	Na-Formate 1.5 M (mL)	Na-Formate Pd Mole Ratio	pH	Wt. Gain (%)	t_1/2_ (min)	T_max_ (°C)
A1	Dil. HCl	2.5	1	18.6	3.8	No wt. gain	No activity was observed.	
A2	Dil. HCl	2.5	10	186.2	6.9			
A3	Dil. HCl	2.5	2	37.2	4.0			
A4	SnCl_2_ + PdCl_2_	2.5	1	18.6	3.8			
A5	SnCl_2_ + PdCl_2_	2.5	10	186.2	6.7			
A6	SnCl_2_ + PdCl_2_	2.5	2	37.2	4.1			
A7	Oxalic acid	2.5	1	18.6	3.7	0.09		
A8	Oxalic acid	2.5	1	18.6	4.0	0.07		
A9	Oxalic acid	2.5	2	37.2	4.1	0.96		

**Table 2:** Details of palladium-based catalysts over 4 cm × 6 cm SS wire gauge preparation using formaldehyde as a reducing agent.

No.	Activation	PdCl_2_ (mL) (Pd = 3.6 mg/mL)	Formaldehyde 1:10 (mL)	Formaldehyde: Pd Mole Ratio	Weight Gain (%)	t_1/2_ (min)	T_max_ (°C)
B1	Dil. HCl	36	18	21	2.1	No activity	
B2		36	18 + 18	42	3.0		
B3		36	36	42	3.3		
B4		11.3	6	20	0.86		
B5		22.6	12	20	2.4		
B6	SnCl_2_ + PdCl_2_	25	6	10	2.3		
B7	SnCl_2_ + PdCl_2_	25	12	20	2.3		
B8	Oxalic acid	36	18	21	2.3	6.5	220
B9	Oxalic acid	36	18 + 18	42	3.6	6.5	310
B10	Oxalic acid	18	9 + 9	42	2.1	6	250

**Table 3:** Details of palladium-based catalysts over 6 cm × 8 cm SS wire gauge coupons.

No	PdCl_2_ (mL) (Pd = 3.6 mg/mL)	Formaldehyde (1:10 (mL)	Formaldehyde/Pd Mole Ratio	Coating Time (h)	Weight Gain (%)	t_1/2_ (min)	T_max_ (°C)
C1	36	36	42	7–8	2.01	3.5	232
C2	36	18 + 18	42	9–10	2.07	3	315
C3	36	54	62	8	1.93	3.5	275
C4	36	72	82	7	2.16	3.5	200

**Table 4:** Details of platinum-based catalysts over 6 cm × 8 cm SS wire gauge support.

No	Activation	A = H_2_PtCl_6_ (Pt = 9.5 mg/mL),	Formaldehyde (1:10) (mL)	Formaldehyde/Pt Mole Ratio	Coating Time (h)	Weight Gain (%)	t_1/2_ (min)	T_max_ (°C)
D1	HCl (0.1 N)	6.3	9.8	41	9	1.7	5	248
D2	Oxalic Acid	6.3	9.8	41	20	1.81	5.1	275
D3	HCl (0.15N)	6.3	9.8	41	8	1.58	4.8	257
D4	Oxalic Acid	6.3	14.8	62	10	1.65	5.1	270

**Table 5:** Details of sequential and simultaneous coating of platinum and palladium.

No	Precursor (mL)	HCHO (1:10) (mL)	HCHO/ Pd, Pt Mole Ratio	Coating Time (h)	Weight Gain (%)	t_1/2_ (min)	T_max_ (°C)
^#^ E1	H_2_PtCl_6_ = 3.2	5	42	9	0.46	---	
	PdCl_2_ = 17	18	42	10	2.08	2.8	295
^#^ E2	H_2_PtCl_6_ = 3.2	12	100	9	0.41	---	
	PdCl_2_ = 17	18	42	10	2.08	2.6	290
^#^ E3	H_2_PtCl_6_ = 4.7	7.6	43	9	0.87	---	
	PdCl_2_ = 17	18	42	10	2.14	2.8	300
* F1	PdCl_2_ = 17 H_2_PtCl_6_ = 3.2	36	65	9	1.3	Not observed	
^#^ F2	PdCl_2_ = 17 H_2_PtCl_6_ = 3.2	36	65	9	1.2	2.6	338
^#^ F3	PdCl_2_ = 17 H_2_PtCl_6_ = 3.2	36	65	9	1.3	2.4	313

^#^ Etched with HCl, * etched with oxalic acid, Pd = 3.6 mg/mL, Pt = 9.5 mg/mL/.

## Data Availability

Not applicable.
